# Waterlogging Tolerance of Crops: Breeding, Mechanism of Tolerance, Molecular Approaches, and Future Prospects

**DOI:** 10.1155/2013/963525

**Published:** 2012-12-24

**Authors:** F. Ahmed, M. Y. Rafii, M. R. Ismail, A. S. Juraimi, H. A. Rahim, R. Asfaliza, M. A. Latif

**Affiliations:** ^1^Department of Crop Science, Faculty of Agriculture, Universiti Putra Malaysia, 43400 Serdang, Selangor, Malaysia; ^2^Institute of Tropical Agriculture, Universiti Putra Malaysia, 43400 Serdang, Selangor, Malaysia; ^3^Bioscience and Agrotechnology Division, Malaysian Nuclear Agency, Bangi, 43000 Kajang, Selangor, Malaysia; ^4^Rice and Industrial Crop Research Centre, Malaysian Agriculture Research and Development Institute (MARDI), Locked Bag No 203, Kepala Batas Post Office, 13200 Seberang Perai, Pulau Pinang, Malaysia; ^5^Plant Pathology Division, Bangladesh Rice Research Institute (BRRI), Gazipur, Dhaka, Bangladesh

## Abstract

Submergence or flood is one of the major harmful abiotic stresses in the low-lying countries and crop losses due to waterlogging are considerably high. Plant breeding techniques, conventional or genetic engineering, might be an effective and economic way of developing crops to grow successfully in waterlogged condition. Marker assisted selection (MAS) is a new and more effective approach which can identify genomic regions of crops under stress, which could not be done previously. The discovery of comprehensive molecular linkage maps enables us to do the pyramiding of desirable traits to improve in submergence tolerance through MAS. However, because of genetic and environmental interaction, too many genes encoding a trait, and using undesirable populations the mapping of QTL was hampered to ensure proper growth and yield under waterlogged conditions Steady advances in the field of genomics and proteomics over the years will be helpful to increase the breeding programs which will help to accomplish a significant progress in the field crop variety development and also improvement in near future. Waterlogging response of soybean and major cereal crops, as rice, wheat, barley, and maize and discovery of QTL related with tolerance of waterlogging, development of resistant variety, and, in addition, future prospects have also been discussed.

## 1. Introduction

In the world of increasing food demand scientists are trying to produce more crop in the barren farmland and in unfavorable condition. Usually in those areas water, soil condition, and temperature are not favourable for agricultural production. Waterlogging is one of the most hazardous natural occurrences, which can also be called as flood, submergence, soil saturation, anoxia, and hypoxia, which are generally used to describe waterlogging conditions depending upon the moisture or water level on the field. Generally, two types of flooding are present in the field: (1) waterlogging, in which root and some portion of the shoot goes under water, and (2) complete submergence, where the whole plant goes under water. 

Landraces cultivated by local farmers include varieties adapted to waterlogging or complete submergence which could be used as a source of genetic material for improving the tolerance of rice to some environmental stresses like waterlogging. Almost a total of over 22 million hectares of lowland rice cultivable area worldwide are vulnerable to flash flooding, representing a total of almost 18% of the global supply [1]. 10 million hectares of land in India and Bangladesh are regularly affected by flood in monsoon season regularly [2]. To avoid such damage, farmers traditionally cultivate flood-adopted lowlands and landraces which can withstand complete submergence or flooding for 10 days or more and can resume its growth after desubmergence [3]. Usually these landraces have total yield not more than 2 t/ha, in contrast to semidwarf varieties which have yield of 6–8 t/ha. Regrettably, the “mega varieties” which are grown widely in different parts of Asia are prone to flooding and dies within one week. Plant breeders identified this problem in the 1970s, while trying to increase production in the low-lying areas by introducing submergence tolerance traits to high-yielding varieties which are popular to local farmer [4].

 Lowland rice varieties are usually cultivated in fields with standing water of 5–25 cm, usually vulnerable to seasonal flash floods of about 50 cm or more water level which can completely submerge entire field of crops. Frequently, stagnant flooding also occurs due to heavy rainfall with flash flood and deepwater flood. In case of flash flood plant restricts its growth when under water and after removal of water growth is restored. In deepwater flood, most of the rice variety show unique capacity of elongation of leaves and some part of stem (internode) which is entrapped under water. After flood this elongated tall plant easily lodges when flood water moves away. In case of deep water flood, underwater elongation could cause death of the plant within few days by exhausting reserve energy of the plant.

In case of barley, waterlogging causes chlorophyll, protein, and degradation of RNA, and also reduces the concentration of nutrients like nitrogen, phosphorus, metal ions, and minerals in shoot. After the onset of flood leaf chlorosis starts [5–7], root and shoot growth was also affected which results in reduction of accumulation of dry matter and also finally in yield [8–11]. Estimated 20–25% yield loss can happen depending upon plant damage; it may be exceed 50% due to waterlogging [12]. 

Maize is also susceptible to waterlogging which causes loss of yield in tropical and subtropical region. Fifteen percent of all maize growing areas of South-East Asia face waterlogging problem, which may lead to yield loss of a range about 25–30% annually [13].

Damages of soybean because of waterlogging are chlorosis, necrosis, stunting, defoliation, reduced nitrogen fixation, and plant death which causes yield loss [14–18] and all of these symptoms occur at various vegetative and reproductive stages of the plant [16, 18–21]. Only 2 days of flooding can cause 18% of yield loss at late vegetative stage while it may exceed to 26% if flooding occurs at early reproductive stage of soybean [19]. Flooding regularly affect soybean growth and grain yield around the world including USA [21, 22]. 

Waterlogging is a major problem for wheat cultivation around the world and in USA where around 12% of cultivated soil affected by excess water [23]. About 39-40% yield loss is recorded under waterlogging condition [24, 25]. Researchers suggested that it is a combining effect of reduced kernel and tiller numbers [24] which is responsible for reducing yield of wheat in waterlogging.

## 2. Mechanism of Waterlogging Tolerance

Waterlogging is a serious problem in low-laying rainfed areas. Lack of oxygen supply for the plant is the main reason of damage in waterlogging condition, which hampers nutrient and water uptake, as a reason the plant shows wilting. In oxygen-deprived condition plants shift its metabolism to anaerobic from aerobic mode. Plants which can withstand waterlogging condition have mechanisms such as increased availability of soluble sugar, aerenchyma formation, greater activity of glycolytic pathway and fermentation enzymes, and involvement of antioxidant defense mechanism to cope with the oxidative stress induced by waterlogging. Ethylene plays an important role in change of mechanisms of plants in deficiency of oxygen. It was also reported that ethylene induces the genes of enzymes associated with aerenchyma formation, glycolysis and fermentation pathway [26].

### 2.1. Hypoxia and Anoxia

 Roots of the plants are usually in contact with oxygen at a partial pressure which is equivalent to the gaseous atmosphere. Hypoxia, reduction of oxygen below optimum level, is the most common form of stress which occurs during partial submergence of plant due to short-term flooding where the root goes under water and shoots remain in the atmosphere. Anoxia, another kind of water stress where plant goes under water completely, hence complete absence of oxygen as a result of long-term flooding. Microbial flora of the soil may change by long time waterlogging which works in favour of anaerobic microorganisms that use, as alternative, electron acceptors to oxygen. In such condition soil tends to accumulate nitrite as it tends to accumulate more reduced and phytotoxic forms of mineral ions (from nitrate) and ferrous ions (from ferric) and very few number of plants are naturally adapted to grow in this kind of soil [27].

### 2.2. Ethylene Production and Flooding

 Accumulation of ethylene in waterlogged soil and plants occurs at concentration of 10 cm^3^ dm^−3^ [28]. This accumulation of ethylene occurs in mainly two ways, firstly ethylene diffusion rate from root to water is 10 times slower than in air [29] and, ethylene synthesis is increased in the hypoxic root system and in the aerobic shoot [30]. At first ethylene may be released to the internal channels of aerenchyma and then diffused to the root area. In roots high amount of ACC (1-amino cyclopropane 1-carboxylic acid) synthesis is occurred, which is a precursor of ethylene [31]. ACC to ethylene conversion cannot happen without oxygen; as a result this reaction is blocked in the aerobic root system [26]. Formation steps of ACC do not require oxygen, furthermore, accumulation of ACC rather increased in aerobic condition [32]. As a result the amount of ACC is increased to the shoot. Highest amount of ACC accumulation occurs in the lowest portions of the stem and ethylene is released in the presence of oxygen. In partial submergence rice undergoes through a special pathway initiated by the entrapment of ethylene in the plant cell. As a result ethylene encourages degradation of abscisic acid (ABA) and increases the content of gibberellic acid (GA) and their downstream effects [33, 34]. Remarkably, the rates of stem elongation in deepwater rice varieties may be up to 25 cm/day. This phenomenon of deepwater rice is mainly controlled by three QTLs. Of these, the SNORKEL QTL on chromosome 12 encodes two ethylene responsive factor (ERF) DNA binding proteins, SNORKEL1 (SK1), and SNORKEL2 (SK2), which were absent to nondeepwater rice accessions [33].

### 2.3. Role of Ethylene in Aerenchyma Formation

Formation of aerenchyma is initiated by ethylene, which is one of many adaptive features of plants at submergence to avoid anaerobiosis by increasing the availability of oxygen. The soft tissues with large intercellular spaces to render low-resistance internal pathways for the exchange of gases between aerobic shoot to the anaerobic root are called Aerenchyma [35]. This feature has been reported in many species like, *Trifolium subterraneum* [36], wheat [37], rice [38], barley [39], maize [40], soybean [15], *Vigna luteola,* and *Carex* spp. [41, 42]. Oxygen leaks out into the roots and surrounding soil through the aerenchyma. As a result, a small oxygenated soil environment formed which might create aerobic environment to microorganisms and prevent the development of potentially toxic soil components such as oxides of Iron, Cu, and Mn [43]. Synthesis of ethylene was highly increased in roots under hypoxia condition, simultaneously with the formation of aerenchyma [44].

### 2.4. Role of Ethylene Accumulation in Adventitious Roots Formation

Portion of the plant stem in flooded condition produces adventitious roots and grows horizontally (diageotropism). This may be the new roots which replace old root systems [45]. Because of the position of the new roots is close to water surface and they are connected to the stem, close to the formatted aerenchyma, they have more access to oxygen than the old root system. Large air space between these roots enables diffusion of gas between roots and shoots. Adventitious root primordia of deep water rice initiates as a normal plant development but the formation is initiated by the death of nodal epidermal cell covering the tip of primordia which occurred as a result of flood-induced ethylene development [46]. This helps adventitious root development and prevents injury to the growing root. 

## 3. Conventional Breeding for Tolerance

A good number of researches have been done to find out the morphological, physiological, anatomical, molecular, biochemical response of plant to anoxia and hypoxia for at least two decades [47–51]. Lack of oxygen has been suggested as main difficulty for plant [52], during the time and after the flooding plant may be affected by root rot disease which cause reduction of growth and loss of yield [52], also includes nitrogen deficiency of plant [53], or nutrient imbalance [54–56]. 

 Crops in different low-laying areas were subjected to flooding for various periods. Few landraces and traditional varieties of rice have the mechanism to withstand flooding which was first recognized in 1950s and screened in 1970s. Indian accessions FR13A and FR43B and some Sri-Lankan varieties like, Kurkaruppan, Goda Heenati, and Thavalu have been known for their tolerance against complete submergence while FR13A was found to be 100% tolerant for 7 days of complete submergence of 10 days old seedlings [4, 57, 58]. Because of some poor agronomic characteristics like, photoperiod sensitivity, low yields and poor quality of grain, and tallness, FR13A needs to be crossed to produce submergence tolerant variety as a donor for submergence. Effort of producing such a variety started in 1980s [1, 59, 60] and in mid 1990s a submergence tolerant IR49830- 7-1-2-2 of FR13A-derived breeding line was produced and the submergence tolerant gene was introduced to high yielding short-to-intermediate statured lines [61, 62]. It was found that submergence tolerance was controlled by a single or few major genes with some minor genes with smaller effect [1]. 

However, because of their lodging-proneness and susceptibility to insect, pests, and disease these traditional-submergence tolerant varieties are not suitable for large scale production. For this reason IRRI tried to develop submergence tolerant variety with desirable characteristics. “IR 49830-7-2-2” is such a line which is high yielding and resistant to pest and disease and has been used as donor parent; India developed “Sudhir” which is derived from “FR13A” × “Biraj” crosses [63]. Despite the above achievements, till date no desirable submergence tolerant variety with high yield has been developed. 

Other major attempts include development of two population of DHL by IRRI using double haploid lines (DHL) by using cross between submergence tolerant and intolerant cultivars [64]. Intermediate height high-yielding rice varieties like “Jagannath” (OUAT) and “Pankaj” (IRRI) in India in 1969 and “Mahsuri” in Malaysia in 1971 was a landmark in lowland rice breeding. In early 80's two varieties “Savitri” and IR42 developed by Central Rice Research Institute (CRRI) and IRRI, respectively, proposed as high yielding and resistant to insect, pests, and diseases but not as tolerant to submergence as FR13A. Nowadays, scientists are using these varieties as a base material for the development of submergence tolerant variety.

Nowadays accumulated CO_2_ in the root zone of soybean plant has been considered as the main reason of injuries of plant. Breeding for flooding tolerant soybean cultivar aims at developing a variety which can give maximum grain yield from a flooded field where as other attributes such as plant height, root and shoot biomass, leaf colour, and so forth are also considered as major determinants [17]. Application of that knowledge to produce flooding tolerant soybean variety is not yet successful which may be very useful for growers. Genetic viability for soybean flood tolerance has been discovered [18], marker assisted selection (MAS) has been successfully used to breed crops with genetically improved QTLs [65].

Various species of wheat were studied under continuous flooding conditions to determine difference of growth and yield [66]. Waterlogging tolerant cultivars of wheat were identified [24] which unlocks the potential of waterlogging tolerant variety research of wheat. Presence of *Adh* gene in wheat which is also found in barley and rice are associated with waterlogging tolerance to ensure the presence of tolerance mechanism in wheat. All possible crosses between waterlogging tolerant cultivars of spring wheat were made and suggested that waterlogging is controlled by a few number of genes [67]. Another experiment was conducted to find out the narrow-sense of heritability for grain yield and yield components of 80 families of soft red winter wheat population of F_2_ in waterlogging condition to provide selection criteria for further breeding. The plant was kept on five week of waterlogging and REML (Restricted Maximum Likelihood) was used to find out the genetic variance components. Kernel weight was found to be the highest heritability (0.47) along with chlorophyll content (0.37), and tiller number (0.31) and the lowest heritability were grain yield (*h* ^2^ = 0.25) attributes. Kernel weight and grain yield was found to be highly correlated. Selection for a relatively highly heritable trait, as kernel weight, would be an effective way to improve waterlogging tolerance in early generations, as grain. Total yield increase of 17% could be done by selecting on the basis of the index: grain yield-kernel weight-tiller number [68].

## 4. Marker Assisted Breeding for Submergence Tolerance

Since in mid 1990s major genes responsible for waterlogging tolerance have been identified (Table 1) and it become easy for the researcher to concentrate on modification or use of those genes to develop new waterlogging tolerant crop varieties like rice, wheat, maize, soybean, barley, and so forth. In the case of rice introgression of *Sub1 *gene to specific varieties by marker assisted backcrossing (MAB) for various land types and choice of the farmer and addition of new varieties through genetic engineering became possible.

### 4.1. Identification of QTL Associated with Waterlogging Tolerance

Genetic control of submergence tolerance was unknown until mid-1990s. It was suggested as a quantitative trait [1, 59, 74, 75]. *Sub1* the major QTL responsible for submergence tolerance was found out by molecular mapping on chromosome 9, which contributes almost 70% of phenotypic variation in tolerance [76]. Furthermore, it was also suggested that the major chromosome 9 along with other minor QTL is not responsible for more than 30% of the submergence tolerance of rice [77–79]. *Sub1 *was mapped to a 0.16-cM region with circa 3,000 F_2_ progeny on 9th Chromosome [80] and using 4,022 F_2_ individuals a fine-scale physical mapping of *Sub1* also done, which further specified the position of locus 0.075 cM [69] using two different types of varieties, the resistant indica Teqing and tolerant FR13A derivative IR40931-26. 

Detail analysis of ERFs related to *Sub1* locus indicates the duplication of *Sub1*B leads to the development of *Sub1*A which may happen after the *indica* rice domestication [81]. Based on variations of nucleotide in the protein coding region the *Sub1*A is divided in two allelic forms, for example, *Sub1*A-1 and *Sub1*A-2 in submergence tolerant and intolerant accessions of *indica* and *aus *lines [69]. FR13A consists of *Sub1*A-1 which is only found in tolerant varieties, on the other hand, *Sub1*A-2 allele could be found in nontolerant *indica *accessions. Both of the allels encode identical proteins. The difference is, intolerant allele contains Pro186 and, in the tolerant allele it is Ser186. In case of expression, *Sub1*A-1 promotes rapid, prolonged, and pronounced transcript accumulation in leaves of 2- to 4-weeks-old plants in case of submerged condition; on the other hand *Sub1*A-2 promotes a lower level of transcript induction [69, 82]. Detailed analysis of different rice accessions with different* Sub1 *haplotype showed that submergence tolerance is associated with variable levels of *Sub1*A transcript in internodes and nodes at the heading stage of the plant [83]. 

For the identification of QTL associated with waterlogging tolerance of barley different parameters was set like leaf chlorosis, plant survival rate, and biomass reduction. Then compare the QTLs identified in two seasons in two different populations using a composite map prepared by the analysis of different molecular markers like SSRs, RFLP, and DArT (Diversity Array Technology). In two barley double haploids (DH) population twenty QTLs associated with waterlogging tolerance was identified and they were validated by different replication of experiments or by different location trials. It was suggested that most of the QTLs were associated with leaf chlorosis and plant survival while some of them affected multiple waterlogging related traits.Such as QTL, Qwt4-1 contributed to overcome different waterlogging related stress like leaf chlorosis and reduction of plant biomass [70]. 

Two maize inbreed lines were crossed for the purpose of determining waterlogging tolerance, that is, “HZ32” (highly waterlogging-tolerant) and “K12” (highly waterlogging sensitive) [84, 85] were crossed for developing F_2_ mapping population. In the maize growing season of years 2004 and 2005, two experiments were conducted, namely, EXP.1 and EXP.2, respectively, with the same F_2:3_ families along with parents and F_1_ hybrid with a treatments of controlled (without flooding) and waterlogging. One hundred seventeen SSR markers with average space of 11.5 cM were used for the preparation of F_2_ population genotyping which was a base-map of 1710.5 cM length. By the use of composite interval mapping (CIM), QTL associated with different plant characters like, root dry weight, root length, plant dry weight, plant height, shoot dry weight, and waterlogging tolerance coefficient were identified in both the experiments, EXP.1(2004) and EXP.2 (2005) in control and waterlogging condition, respectively. In EXP.1, 25 and in EXP.2, 34 QTL were identified where the effects of discovered QTLs were moderate with a range of 3.9–37.3%. Some major QTLs were identified in two chromosomes 4 and 9 and in both experiments associated with root dry weight, shoot dry weight, total dry weight, plant height, and their waterlogging tolerance coefficient. Chromosome 1, 2, 3, 6, 7, and 10 was also identified [71] as secondary QTLs associated with tolerance. 

To determine the QTL associated with waterlogging tolerance of soybean, two hundred eight lines of two recombinant inbred (RI) populations, “Archer” × “Minsoy” and “Archer” × “Noir I”, were put into two different experimental setup, that is, one with control (without waterlogging) and other with waterlogged condition, and the plants of waterlogged setup were put into two weeks of flooding at early flowering stage. A single QTL was identified which was responsible for better plant growth and grain yields, 11–18% and 47–180%, respectively, in waterlogging environment linked to marker sat_064 from the parent Archer [72]. In another experiment two populations, that is, A5403 × Archer (Population 1) and P9641 × Archer (Population 2) were used to identify QTLs associated in waterlogging tolerance where 103 and 67 F_6:11_ RI lines, respectively, were used for mapping population. The performance of both the populations was tested on the flooded field setup and significant variations were observed, however, no transgressive tolerant segregants were found in both populations. Whole sample was divided into sensitive and tolerant bulk for the purpose of further molecular study.

In molecular genetics studies, two methods, named, SMA (single marker analysis) and CIM (composite interval mapping), were used for the identification of the QTL associated with waterlogging tolerance. Out of 912 SSR markers, 17 markers (population 1) and 15 markers (in population 2) were found associated with waterlogging tolerance in SMA. Most of the markers were closely related with *Rps* gene or QTL responsible for the resistance to Phytophthora (*Phytophthora sojae* M. J. Kaufmann and J. W. Gerdemann). In CIM five markers, that is, Satt59, Satt160, Satt269, Satt252, and Satt485 were found associated with waterlogging tolerance [73] in both populations. This analysis also suggested that most of the genes come from the parent Archer. 

### 4.2. Manipulation of QTL for Developing Waterlogging Tolerant Variety

Flooding is a natural calamity that destroys production of rice throughout the world especially in low-laying areas, but all economically important varieties are not tolerant to flooding. Identification of the SUB1 QTL enables the scientist to introgress this gene by MAB into the popular high-yielding varieties [86, 87]. Mapping of the Sub1 allele reveals SNP (single-nucleotide polymorphisms) within *Sub1A* and *Sub1C, which* is useful for the breeders for molecular markers and in precision breeding [88, 89]. By the use of MAB, *Sub1A* has been already inserted to modern varieties of different countries; BR11, Swarna, Samba Mahsuri IR64, CR1009 and Thadokkam 1 (TDK1) are some of the examples of those varieties [88]. SSR markers, which were polymorphic between two parents, were generally used for the background conformation or recurrent parent genome conformation also well combined with the Sub1 region originated from FR13A on chromosome 9. Normally newly developed Sub1 lines shows more significant waterlogging tolerance compared to parents [88, 90] which is find out by evaluation. These studies show the opportunities of the insertion of the Sub1 region from developed tolerant variety through MAB to produce tolerant varieties with a diverse genetic background. Furthermore, the effect of *Sub1* on other agronomical characteristics of plant such as, grain quality, growth, maturation, and grain production was determined in IR64 Sub1, Swarna Sub1, and Samba Mahsuri Sub1. Further research of the above mentioned submergence tolerant varieties and their original parents revealed that insertion of *Sub1* gene does not alter field performances including quality and yield of grain under normal growth condition [83, 89, 91]. 

Complete waterlogging of susceptible varieties at the different growing stage considerably reduces the yield attributes like, number of panicles, total number of grains per panicle, and grain filling percentage of the plant and also flowering and maturity may be delayed, resulting in a remarkable decline in yield. On the other hand, a Sub1 rice variety minimizes the total yield reduction by flood and produces more yield than the intolerant varieties at submerged condition [83].

 One of the main advantages of using this approach is that the newly developed Sub1 varieties retain almost all agronomical characteristics of the recurrent parent, especially in terms of yield and quality. Sub1 varieties produced from FR13A-derived varieties have almost the same yield, agronomical, and grain quality characteristics as recurrent parent varieties when grown under regular condition, but, when subjected to flooding for 1 to 2 weeks, Sub1 varieties showed a remarkable advantages in terms of yield than the susceptible ones [83, 90]. Mega varieties which has *Sub1* gene, can be adopted by the farmer easily, in addition, these new varieties can replace the traditional landraces with low yielding, currently which has been used by the farmers in flood prone areas. 

Some tolerant lines were evaluated and adopted in low-lying areas in more than ten countries in South and Southeast Asia, in field trials as the preferences of the farmers [92]. The good yield performance of newly developed varieties, showing the better performance of *Sub1* against flooding or submergence of rice. Those performances of the newly developed varieties have influenced a lot of rice improvement programs in Asia and Southeast Asia to perform rapid seed multiplication and dissemination schemes. 

## 5. Improvement of Flooding Tolerance in Rice through Plant Genetic Engineering Techniques

Current molecular biology and biotechnology methods for rice research have been improved to such level that rice has been considered as a model crop in cereal breeding. Transformation of gene of rice was firstly done by protoplast-based method through using microprojectile gun, and with the advance of technology with time *Agrobacterium *mediated gene transfer of rice is also available. The regulatory sequences of controlling expression of transgenes in rice are available now, so as a result, the transgene can be either normally expressed or expressed in response to a specific stimulus or artificially (including induced anaerobic stress, through the use of promoters from genes which strongly respond to waterlogging). Transgene rice with specific attributes like, tolerance to virus, insects. Pests, salt, low temperature, and flood can be produced [93]. Identification/isolation/cloning of associated genes with waterlogging stress considered as limiting factor [93], as a result scientists focused on carbohydrate metabolism because reduced O_2_ supply hampers normal respiration resulting in decrease in ATP synthesis. It has been reported that, respiration pathway switch to fermentation pathway from oxidation during oxygen-deprived (anaerobiosis) condition. The inclusion of ethanolic fermentation pathway is considered to be an important component of responses which are elicited in rice (and other plants) against flooding stress [93, 94]. Ethanolic fermentation, that is, pyruvate to ethanol is a relatively simpler process involving two enzymes, that is, PDC or pyruvate decarboxylase and ADH or alcohol dehydrogenase. Availability of cloned *pdc* and *adh* normally attracts the interest of molecular biologists to use these for transgenic experiments. Researchers at CISRO, Australia are trying to alter the different levels of PDC and ADH in rice and as a result 3 different *pdc* genes in rice, that is, *pdc1, pdc2,* and *pdc3* have already been cloned and sequenced. *pdc1 *cDNA has been subcloned at the 3′ end of three different promoters that is, CaMV 35S, actin 1 and anoxia-induced 6X ARE promoter, which is a synthetic promoter, in both sense and antisense orientations and these plasmid constructs have been introduced into rice to produce a large number of transgenes [95–97]. One of the above gene constructs (actin 1-pdc1, sense) has been employed at IRRI for the production of transgenic rice with enhanced metabolic capacity under anaerobiosis conferring submergence tolerance [98]. In the study, tillers of transformed plants show higher *pdc* activities and ethanol production than untransformed plants and consequently ethanol production of tillers of transgenic plants was positively correlated with survival after submergence. Scientist of CISRO also made considering progress to change in the ADH level in rice [99]. However, no attempts directed at making transgenes by using those have been taken.

## 6. Conclusions and Future Prospects 

Combination of both MAS and traditional field selection could increase the efficiency of barley breeding programs. Now, it is suggested that diversity array technology (DArT) is most useful for genome profiling [100]. DArT markers can be used for the analysis of waterlogging tolerant genes or QTLs in grass families, and in future researches it may be possible to make comparison of a DNA sequence to the genome sequence of rice and other species [101, 102]. 

Physiologies of rice plant under submerged condition have been studied intensively since the work of Yamada [103]. The damage caused by waterlogged condition may be associated to the associated action by water in gas exchange and illumination. Reaction of the plats to avoid such damage caused by impeded gas exchange includes an increase of leaf elongation, stopping photosynthesis, and leaf senescence. These actions may be taken by the plant to decrease energy demand of the plant to maintain cell integrity in important tissues like meristematic region of leaf bases, root tips, and stem. Survival mechanism of rice plants against submergence became clearer. It is proposed that tolerance of a plant is permitted by minimizing the imbalance between production of energy and its usage. This is promoted by a suppression of the high rates of leaf elongation that submergence normally does. Furthermore, reduction of leaf senescence during submergence has been implicated. Elongation of leaves in waterlogged condition and senescence could be implicated by ethylene which is trapped within the plant cell because of its diffusive escape which is more delayed by water then air. These ethylene-induced effects are suppressed in tolerant lines. Researchers also find out that presence of *Sub1* locus on chromosome 9 of rice which have act upon senescence and leaf elongation, effects of injury, and survival. This effect is dominant, that is, effective in the heterozygote carrying only one FR13A-derived segment [78] and may affect ethylene responsiveness of the plant. It is one of the major challenges for scientists to modify the effects of environmental stress [97, 104]. Tightly linked DNA markers gives opportunities for transferring the submergence tolerance characteristics from FR13A or other lines into economically important cultivars. Such markers are known to be carried over when tolerance is introduced into a desirable agronomical background by conventional breeding and progeny phenotyping [78]. Identification of other mechanisms of tolerance derived from resilient cultivars other than FR13A (e.g., Goda Heenati and Vaidehi) remains unknown and represent a promising additional resource for breeders. 

REML method could be very useful in wheat waterlogging tolerance breeding. Breeding programme selecting combination of different criteria including grain yield, kernel weight, and so forth instead of only one character could be more useful to develop high-yielding variety. The total gain from each selection should be confirmed prior to taking steps which lead to the development of new variety [68].

First work about submergence tolerance of maize seedling was done by Mano et al. [105]. Several OTLs associated with waterlogging tolerance with genome-wide significance has been identified which may be useful to clarify the understanding of maize waterlogging tolerance mechanism [71]. Use of different evaluation method, or population, or combination, of both may enables the researchers to detailed understanding of tolerance. Already identified QTLs should be mapped precisely before inserting prior to develop tolerant variety.

Genomic regions responsible for waterlogging tolerance of soybean can be helpful for MAS to develop high-yielding flooding tolerance cultivars. It may be possible to combine multiple favourable alleles into one cultivar. Variety selection to use as rotation of soybean and in low-yielding areas vulnerable to waterlogging conditions will be benefited by significant QTLs tolerance information. Further study should be carried out about already discovered QTLs before inserting into popular variety through marker assisted selection [73].

Scientist around the world working to produce waterlogging tolerance varieties of different crops, till date the information about QTLs of waterlogging is very limited and needs to be explored.

## Figures and Tables

**Table 1:** QTL identification of waterlogging tolerance in different crops.

Species	QTL/chromosome No./genes	Marker used	Method	References
Rice (*Oryza sativa L.*)	Sub1 (Ch. 9)	SSR	MAS	[69]
Barley (*Hordeum vulgare L.*)	Qwt4-1	SSR	MAS	[70]
Barley (*Hordeum vulgare L.*)	*tfy2.1-1, tfy1.1-2, tfy1.2-1 *	RFLP	MAS	[70]
Barley (*Hordeum vulgare L.*)	*tfy1.1-3, tfsur-2 tfsur-1, tfy1.1-1, tfmas, tfy2.1-2 *	DArT	MAS	[70]
Maize (*Zea mays L.*)	Ch. 4, 9	SSR	CIM	[71]
Maize (*Zea mays L.*)	Ch. 1–3, 6, 7, and Ch. 10	SSR	CIM	[71]
Soybean [*Glycine max *(*L.*)* Merr.*]	*Rps *	SSR	SMA	[72]
Soybean [*Glycine max *(*L.) Merr.*]	*Rps *	SSR	CIM	[73]

^∗^SMA: Single marker analysis, ^∗^CIM: Composite interval mapping, ^∗^MAS: Marker assisted selection.
